# Effectiveness of the Epley’s maneuver performed in primary care to treat posterior canal benign paroxysmal positional vertigo: study protocol for a randomized controlled trial

**DOI:** 10.1186/1745-6215-15-179

**Published:** 2014-05-21

**Authors:** José Luis Ballve Moreno, Ricard Carrillo Muñoz, Iván Villar Balboa, Yolanda Rando Matos, Olga Lucia Arias Agudelo, Asha Vasudeva, Olga Bigas Aguilera, Jesús Almeda Ortega, Alicia Capella Guillén, Clara Johanna Buitrago Olaya, Xavier Monteverde Curto, Estrella Rodero Perez, Carles Rubio Ripollès, Pamela Catalina Sepulveda Palacios, Noemí Moreno Farres, Anabella María Hernández Sánchez, Carlos Martin Cantera, Rafael Azagra Ledesma

**Affiliations:** 1 Centre d’Atenció Primària (CAP) Florida Nord, Institut Català de la Salut, Hospitalet de Llobregat, de Llobregat, Spain; 2 Centre d’Atenció Primària (CAP) Florida Sud, Institut Català de la Salut, Hospitalet de Llobregat, de Llobregat, Spain; 3 Técnico de Salud de Soporte a la Investigación. Unitat de Suport a la Recerca. Direcció d’Atenció Primaria de Costa de Ponent. Gerencia Territorial Metropolitana Sud, Institut Català de la Salut (ICS), Barcelona, Spain; 4 Hospital General de Hospitalet, Hospitalet de Llobregat, de Llobregat, Spain; 5 Departament de Medicina, Equip d’Atenció Primària Barcelona, Universitat Autònoma de Barcelona, Passeig Sant Joan, Barcelona, Spain; 6 Equip d’Atenció Primària Badia del Valles. Servei d’Atenció Primària Vallés Occidental. USR IDIAP Jordi Gol. Direcció d’Atenció Primària Metropolitana Nord, Institut Català de la Salut, Universitat Autònoma de Barcelona, Barcelona, Spain

**Keywords:** Primary care, Canalith repositioning procedure, Benign paroxysmal positional vertigo, Randomized clinical trial, Betahistine

## Abstract

**Background:**

Vertigo is a common medical condition with a broad spectrum of diagnoses which requires an integrated approach to patients through a structured clinical interview and physical examination. The main cause of vertigo in primary care is benign paroxysmal positional vertigo (BPPV), which should be confirmed by a positive D-H positional test and treated with repositioning maneuvers. The objective of this study is to evaluate the effectiveness of Epley’s maneuver performed by general practitioners (GPs) in the treatment of BPPV.

**Methods/Design:**

This study is a randomized clinical trial conducted in the primary care setting. The study’s scope will include two urban primary care centers which provide care for approximately 49,400 patients. All patients attending these two primary care centers, who are newly diagnosed with benign paroxysmal positional vertigo, will be invited to participate in the study and will be randomly assigned either to the treatment group (Epley’s maneuver) or to the control group (a sham maneuver). Both groups will receive betahistine. Outcome variables will be: response to the D-H test, patients’ report on presence or absence of vertigo during the previous week (dichotomous variable: yes/no), intensity of vertigo symptoms on a Likert-type scale in the previous week, total score on the Dizziness Handicap Inventory (DHI) and quantity of betahistine taken.

We will use descriptive statistics of all variables collected. Groups will be compared using the intent-to-treat approach and either parametric or nonparametric tests, depending on the nature and distribution of the variables. Chi-square test or Fisher’s exact test will be conducted to compare categorical measures and Student’s *t*-test or Mann–Whitney *U*-test will be used for intergroup comparison variables.

**Discussion:**

Positive results from our study will highlight that treatment of benign paroxysmal positional vertigo can be performed by trained general practitioners (GPs) and, therefore, its widespread practice may contribute to improve the quality of life of BPPV patients.

**Trial registration:**

ClinicalTrials.gov Identifier: NCT01969513.

## Background

Dizziness, a common complaint in patients presenting to the primary care office and the emergency department, is a disorder of spatial orientation. Approximately 3% of the visits to US emergency departments were accounted for by dizziness presentations according to data from a nationally representative study [1].

Vertigo is a subtype of dizziness, defined as an illusion of motion caused by a mismatch of information from the visual, vestibular and proprioceptive systems. Vertigo is divided into central and peripheral causes. Central vertigo is generally more serious, whereas peripheral vertigo is usually benign. It has been estimated that 45 to 54% of the patients who attend the primary care physician with dizziness are suffering from vertigo [2]. The three most common causes of vertigo (accounting for 93% of all patient presentations) are: acute peripheral vestibulopathy (vestibular neuritis and labyrinthitis), Ménière’s disease and benign paroxysmal positional vertigo (BPPV), the latter being the most frequent [3].

The mean age of onset of BPPV is 49 years, its lifetime prevalence is approximately 2.4%, and its cumulative incidence reaches almost 10% at the age of 80 years [4]. The theories that try to explain the pathophysiology of BPPV are based on clinical and histopathologic findings. BPPV occurs as a result of displaced otoconia, which are small calcium particles (otoliths), normally attached to the otolithic membrane in the utricle. Because of trauma, infection, aging, and even without any known cause, otoliths can detach from the utricle and collect within the semicircular canals [5]. The posterior canal is the most frequently affected of the three semicircular canals. Movement of the head causes these otoliths to inappropriately trigger the receptors in the semicircular canals and send false signals to the brain, causing vertigo and nystagmus, as occurs during the Dix-Hallpike (D-H) maneuver.

The diagnostic approach to vertigo relies on the quality of symptoms reported. Patients suffering from vertigo may be diagnosed by asking the following question: ‘When you are dizzy, do you have a sense that you or your surroundings are spinning or moving?’ An affirmative response makes the diagnosis of vertigo most likely and directs the physician towards a subsequent search for vestibular causes. In the case of BPPV, patients may also present with lightheadedness, unsteadiness, loss of balance, blurred vision, nausea and vomiting, without hearing loss or tinnitus. Abnormal rhythmic eye movements (nystagmus) usually accompany the symptoms of BPPV. Signs and symptoms may come and go, with symptoms frequently lasting from 10 to 30 seconds. Some patients, however, may feel vertiginous for several minutes and the imbalance and nausea may last several hours. The average duration of each episode is two weeks but a third of patients refer episodes longer than a month. Forty-four percent of BPPV cases experience a single episode of dizziness while 56% are recurrent [4]. Activities that bring about the signs and symptoms of BPPV can vary from person to person, but are almost always brought on by a change in the position of the head: turning in bed, neck extension and tilting the head forward. Although rare, it is possible to have BPPV in both ears (bilateral BPPV).

Physical examination is the next and the last step to make an accurate diagnosis. The equipment needed for the physical examination is simple and available in every primary care examination room: stethoscope, otoscope, sphygmomanometer, reflex hammer, tuning fork and a flat examining bed. Coupled with the medical history and physical examination, the Hallpike maneuver (also known as Dix-Hallpike test or) is extensively used in both the diagnosis and short- and long-term control of BPPV. The maneuver is performed on a flat examination table. While the patient is in a seated position, the physician turns the patient’s head 45° to one side, then rapidly, but smoothly lays the patient into a supine position with the head hanging about 20° over the end of the table and observes the patient’s eyes for approximately 30 seconds. The maneuver is repeated with the head turned to the opposite side. The result is positive if the patient develops symptoms (vertigo) and nystagmus. It has a positive predictive value of 83% and a negative predictive value of 52% [3] (Additional file 1: Video 1 Dix-Hallpike maneuver).

Most patients with vertigo have benign disorders and can be successfully managed by the primary care physician. A low percentage of the disorders require laboratory testing, advanced testing or referral to a specialist [6].

Treatment options include watchful waiting, vestibulosuppressant medication, vestibular rehabilitation, canalith repositioning and surgery. Among these treatment modalities, canalith repositioning procedures (CRPs) are the first choice treatment for BPPV. The aim of CRPs is to move the displaced otoliths from the semicircular canal back to the utricle where they belong. Another less effective treatment and more time consuming technique, the Brandt-Daroff exercise, consists of lying down on your side and then getting up quickly. The presumed mechanism for this therapy is to loose and disperse particles from the cupula of the posterior semicircular canal. The aim of this exercise is habituation and compensation of the vestibular system; it does not prevent recurrence and is not always well tolerated.

Out of all the CRPs, the Epley’s maneuver has been the most successfully used, and is particularly indicated in the treatment of posterior canal BPPV. It consists of a series of four quick movements of the head and body from sitting to lying, rolling over, and back to sitting. Each position is maintained for at least 30 seconds or until positional nystagmus ceases [7] (Additional file 2: Video 2 Epley’s maneuver). A meta-analysis of three high-quality clinical trials demonstrated the effectiveness of the Epley’s maneuver in short-term follow-up, measured in terms of the Hallpike test (D-H test) turning negative (odds ratio (OR) = 5.67; 95% confidence interval (CI) 2.21 to 14.56). This meta-analysis concludes that further research in this field should consider the following criteria: 1. The use of a rigorous randomization technique with respect to adequate pre-allocation concealment; 2. The blinding of outcome assessors; 3. The inclusion of a post treatment Hallpike maneuver as a part of the reported results; and 4. Long-term follow-up of patients [8]. Another meta-analysis came to the same conclusions [9]. Little evidence exists to demonstrate any benefit of the Epley’s maneuver in long-term follow-up [10].

This maneuver has proved to be useful in both pediatric [11] and older-aged patients [12] and appears to be safe and effective if the following few contraindications are considered: cervical spinal stenosis, severe kyphoscoliosis, limited cervical mobility, Down syndrome, advanced rheumatoid arthritis, cervical radiculopathies, Paget’s disease, morbid obesity, ankylosing spondylitis, severe lumbar dysfunction and spinal cord injuries. No serious adverse effects have been reported.

The Epley’s maneuver can be performed by general practitioners (GPs). Although most clinical trials on the effectiveness of this maneuver have taken place in specialized clinics, one study conducted in a primary care center demonstrated that trained GPs achieved the same results as the specialists in terms of D-H test turning negative. However, this study could not prove the subjective improvement of the patients compared to the control group [13].

Several authors emphasize the need for more research to be performed in the primary care setting. One article regrets the slow implementation of the results of scientific evidence, highlighting that in Germany only 8% of patients are treated with repositioning maneuvers, and recommending the practice of the D-H and Epley’s maneuvers to these physicians [14]. A study in Israel demonstrated that only 25% of patients with BPPV referred to a specialist had been correctly diagnosed by their doctors and that in most cases, the correct diagnosis had been made by otolaryngologists (ENT specialists). Out of the 120 patients studied, only 2 cases (the 2 submitted by ENT specialists) had undergone the D-H maneuver to reach the diagnosis [15]. A study conducted in Spain found that the average duration between the onset of symptoms and initiation of treatment with canalith repositioning maneuvers in a specialized ENT center was 20 weeks and that only 1 patient out of 60 was correctly diagnosed as BPPV [16]. Moreover, the duration of the illness before receiving Epley’s maneuver was considered the only independent predictor of recurrence according to a long-term follow-up study. A systematic review also recommends that more research be performed and emphasizes that such investigation should include different specialists, other than otolaryngologists or neurologists, such as GPs [17].

Therapy with betahistine dihydrochloride has been widely prescribed in patients with vestibular disorders for symptomatic treatment of vertigo, and especially in Ménière’s disease patients. A meta-analysis undertaken to evaluate the efficacy of betahistine in the treatment of other vertiginous syndromes, such as BPPV (cupulo-canalilithiasis), analyzed seven double-blind, placebo-controlled, randomized studies, and confirmed betahistine’s therapeutic benefit and effectiveness [18]. Several other studies have proved that the combination of betahistine and repositioning maneuvers improve outcomes, in comparison to maneuvers alone [19,20] but its use for BPPV remains controversial [21].

To date, no relevant studies have been performed on the impact of the use of the Epley’s maneuver in primary care settings, in terms of temporary disability (number of episodes and duration), duration of the drug treatment with its subsequent side effects, referral to specialists, number of recurrences and quality of life.

Our clinical trial will be conducted in a primary care setting, and will study the condition at its earliest stages, when patients are more likely to attend our practices, and where the literature is less conclusive. In addition, response to treatment will be evaluated at one month and at one year after inclusion in the study. The effectiveness of the Epley’s maneuver in the remission of symptoms during the first week may reduce the course of vertigo, and may result in improvement of quality of life, temporary disability and decrease in the amount of medication taken.

## Methods/design

### Hypothesis

The Epley’s repositioning maneuver, performed by GPs, is effective in the short, mid and long term for the treatment of BPPV. We expect to find a significant difference of 30% or over in the negativization of the D-H test in the intervention group (Epley’s maneuver) compared to the control group (a sham maneuver), as well as in the clinical improvement of the patients.

### Aim

#### Primary objective

The study’s primary objective is to determine whether the intervention group (Epley’s repositioning maneuver) improves clinically as compared to the control group (a sham maneuver), after a week, a month and a year of follow-up (second, third and fourth visit respectively). Definition of clinical improvement will include negativization of the D-H maneuver, improvement in the subjective perception of vertigo, quality of life and amount of betahistine tablets that the patients report to have taken.

#### Secondary objectives

We will analyze whether there is a statistically significant clinical improvement in the intervention group in comparison to the control group in terms of:

1. Negativization of the D-H test at a week, a month and a year follow-up.

2. Answer to the dichotomous (yes/no) question regarding presence of vertigo in the previous week.

3. Number of new episodes of vertigo between medical visits.

4. Time from the baseline visit to the first new episode.

5. Rate of vertigo severity on a 10 point-Likert scale: 0 = no symptoms of vertigo; 10 = most severe and unbearable.

6. Quality of life measured with the specific questionnaire Dizziness Handicap Inventory- Short form (DHI-S).

7. Amount of betahistine tablets taken, according to the patients’ self-registers.

8. Days of temporary disability due to vertigo or other causes, recorded on the electronic medical record.

### Design

This is a controlled randomized clinical trial, which will be conducted by GPs who will have previously received a two-hour training to perform the repositioning maneuvers under the supervision of an ENT specialist. Patients will be reassessed one week, one month, and one year after the first visit by a different GP from the one who performed the first visit, in order to accomplish blinding of both study participants and personnel.

### Study’s scope

Two urban primary care centers which provide care for a population of approximately 49,400 people.

### Study sample

**Figure 1:**
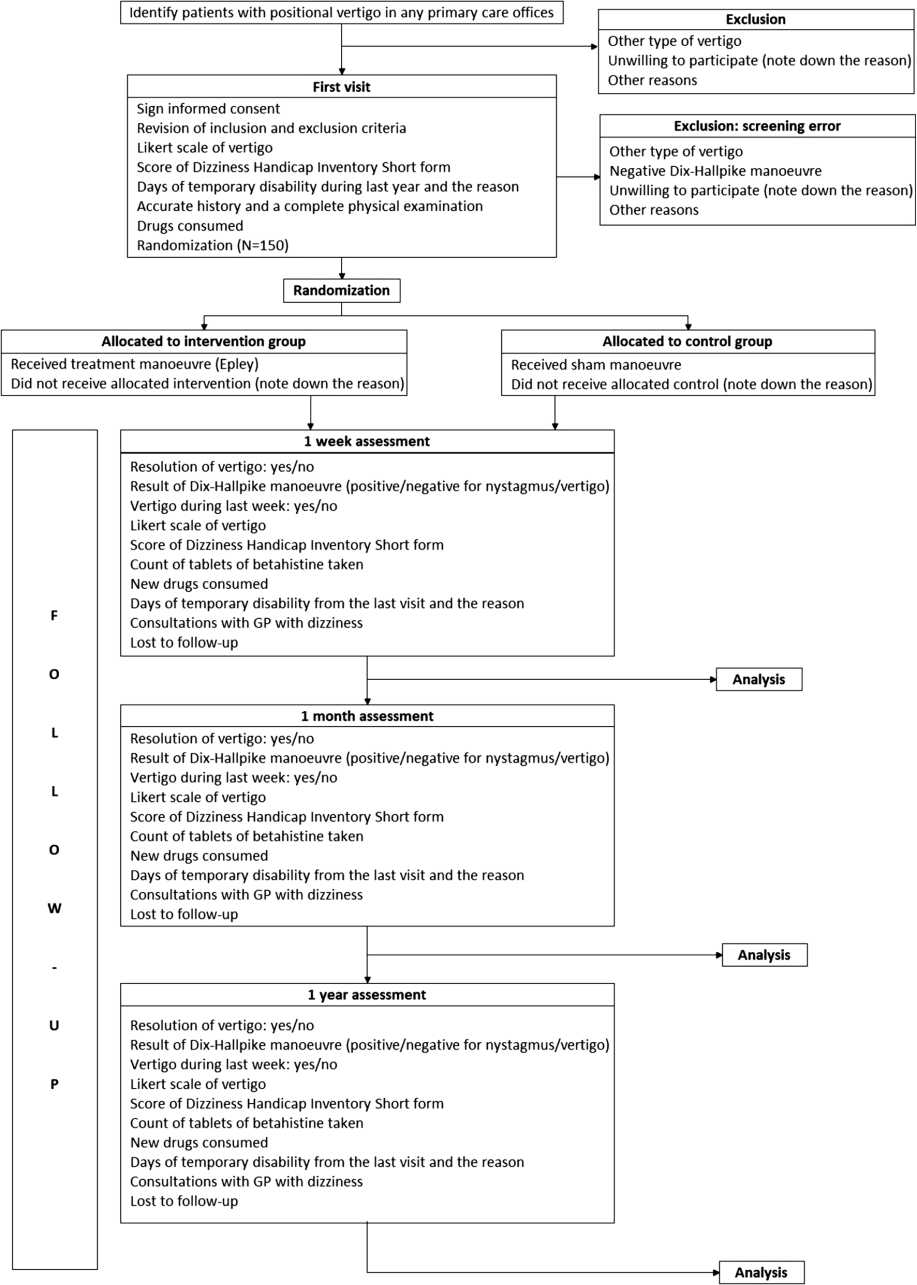
Flow diagram of the study.

All patients with newly diagnosed BPPV, who attend our two primary care centers, will be potential participants in our clinical trial. Patients will be systematically recruited with the collaboration of the 26 GPs who work at the two participant primary care centers. Regardless the initial cause for the visit, if a GP suspects BPPV, he/she will check whether the patient meets the inclusion criteria and presents no exclusion criteria. Eligible participants will be informed of the possibility to take part in the trial and will be supplied with written information about the study. Those who accept to participate in the study will be given an appointment for the baseline visit, preferably within a week and no longer than ten days after. The recruitment period is expected to last two years (Figure 1).

### Inclusion criteria

Patients aged 18 years and older who attend our primary care centers, with suspected diagnosis of BPPV, and present vertigo or nystagmus following the D-H maneuver. All other causes of vertigo should be ruled out through clinical history assessment and review of the electronic medical record. Written informed consent will be obtained from all subjects, of both the intervention group and the control group, prior to their inclusion in the study.

### Exclusion criteria

The exclusion criteria, which will be detected through the clinical history, physical examination and review of the electronic medical record, are:

1. Previous or current diagnoses of labyrinthine diseases such as Ménière’s disease, labyrinthitis or vestibular neuronitis.

2. Contraindications to canalith repositioning procedures: cervical spinal stenosis, severe kyphoscoliosis, limited cervical mobility, Down syndrome, advanced rheumatoid arthritis, cervical radiculopathies, Paget’s disease, morbid obesity, ankylosing spondylitis, severe lumbar dysfunction and spinal cord injuries.

3. Pregnancy or breastfeeding.

4. Contraindications to betahistine administration.

5. Patient refusal to participate in the study.

6. Non-residence in the study area.

7. Other causes which may hinder the understanding of the objectives and methodology of the trial (language, low educational level, and so on).

### Sample size

Accepting an alpha risk of 0.05 and a beta risk of 0.2 in a bilateral contrast, sample size calculations determined a need for approximately 75 subjects in each group (the intervention group and the control group), in order to detect a statistically significant difference in the improvement rate between the two groups (30% for the control group and 55% for the intervention group) at follow-up. We estimated a 20% rate of loss in participants (objectives 1 and 2). This sample size also enables us to evaluate 1-point clinical improvement in the intervention group compared to the control group, assuming a standard deviation of 1.9 (objectives 3,5,6,7 and 8). We estimated a recurrence hazard ratio of 0.5 in the intervention group, assuming that 30% of patients in the control group will have relapsed after one year. The software used for the calculation was GRANMO version 7.12. The software used for the calculation was GRANMO version 7.12 (Program of Research in Inflammatory and Cardiovascular Disorders. Institut Municipal d’Investigació Mèdica, Barcelona, Spain).

### Randomization

All the relevant information will be presented again to the participants at the baseline visit and the opportunity to ask any questions they may deem appropriate will be offered. After signing the informed consent, inclusion and exclusion criteria will be reviewed again and BPPV will be confirmed by the clinical history and physical examination (D-H test positive to the right or the left) at the first visit of the study. Those who meet the following three criteria: presenting no exclusion criteria, all inclusion criteria met, and signed informed consent, will be randomized to the intervention group or the control group. Potential participants who fail to comply with the previous three criteria will be declared ‘screening error’.

Patients will be assigned to the intervention or control group using the randomization sequence list prepared in advance by the study statistician. The responsibility for guarding and supervising the randomization list will rest on a staff member of our primary care center who is not directly involved in the trial. GPs will contact the randomization list guardian by telephone in order to find out the randomization number and to which study arm the participant has been assigned. These data will not be recorded in either the case report forms or in the database. Only the study statistician will be allowed access to this information. Follow-up visits will be carried out by a different GP from the one who performed the first visit in order to accomplish blinding of both study participants and personnel. The randomization software used will be ‘R: A language and environment for statistical computing’, version 2.14.2 (R Foundation for Statistical Computing, Vienna, Austria).

### Interventions

A paper case report form (CRF) has been designed to record all the data from the four visits performed during the trial. Once completed, the CRF will be submitted to the study’s coordinator to enable data introduction in the corresponding database for subsequent analysis. The paper case report form will be reviewed after a pilot test.

### Visit 1: Information and data collection from all participants

All participants will have their electronic medical record reviewed. An accurate medical history will be obtained and a thorough physical examination will be performed. The information collected will include: age, sex, educational level, profession and employment status, date of onset and duration of the symptoms from onset to visit 1, previous history of BPPV episodes (total number), past medical history of other conditions (viral infection in the previous four weeks, head trauma, neck osteoarthritis or neck pain), pharmacological treatment, specially for the treatment of anxiety, depression or hypertension).

During the physical examination we will evaluate blood pressure in the sitting and standing positions, heart rate, color of skin and mucous membranes, cardiac and respiratory auscultation, basic neurological examination (cranial nerves, visual fields, brainstem and muscle stretch reflexes, posture, balance and coordination tests (test station, tests for dyssynergia and dysmetria, and gait), otoscopy and D-H maneuver to the left and right to detect the presence of nystagmus or vertigo [22]. If the examination suggests involvement of the anterior or lateral semicircular canals or presence of a central vertigo (nystagmus that lasts more than a minute, vertical nystagmus or alternant), the patient will be excluded from the study and referred to a specialist.

The results of the D-H maneuver (to the right or left) will be divided as follows:

1. Negative.

2. Positive. This result will be subdivided into: a) vertigo with nystagmus or b) vertigo without nystagmus [23].

The assessment of symptom severity will be performed using a 10-point Likert scale, ranging from 0 = no symptoms of vertigo to 10 = most severe and unbearable.

Assessment of quality of life will be carried out through the Dizziness Handicap Inventory-Short form (DHI-S), in an adapted version translated into Spanish by López-Escamez. The adopted version was validated using the translation-back translation method by two interpreters with clinical experience; both translations were then discussed in a consensus meeting with one of the investigators, yielding to the adapted version, which presents a Cronbach’s alpha internal consistency of 0.8014.

The DHI-S is a 10-item self-assessment inventory effectively used to evaluate the perceived degree of disability caused by vertigo, dizziness and instability and its impact on daily life activities. It also identifies physical, functional, and emotional conditions related to balance disorders [24].

### Visit 1 for patients in the intervention group arm

The Epley’s maneuver will be performed only in the first visit since a single procedure has been shown to improve the condition in 76% of patients [25]. Moreover, most patients may improve spontaneously after a month, regardless of which group they were assigned. The maneuver consists of five sequential positions of the head and body, performed with the aim to move the displaced canaliths from the semicircular canal back to the utricle where they no longer cause symptoms. There is some controversy over whether postural restrictions for a few days after this procedure are beneficial for the patient and can improve outcomes [26]. However, we have decided not to include these restrictions in our study as they are poorly tolerated and can hamper the comparability between groups [27].

### Visit 1 for patients in the control group arm

A sham maneuver, which will consist of laying the patient with the head tilted on the affected side for five minutes, as described in the literature [17,23], will be performed only on the first visit.

### Follow-up visits 2, 3 and 4 to all patients

The second visit will take place 1 week after visit 1. Visits 3 and 4 will be performed 1 month, and 1 year after visit 1, respectively. Telephone reminders will be used to reduce patients’ loss during follow-up.

Follow-up visits will include:

•Bilateral D-H maneuver. Outcome measures will be: nystagmus (yes/no) and vertigo (yes/no).

•Assessment and record of number of new episodes of vertigo, and time (in days) from the baseline visit to the first new episode. This information will be obtained through an accurate medical history, and review of the patient’s electronic medical record, including visits to the emergency department, in our medical centers as well as in other emergency centers. Outcome measures will be: vertigo (yes/no), number of episodes, and intensity of symptoms on a 0 to 10-point Likert scale.

•Registration of the total score on the Dizziness Handicap Inventory-Short form (DHI-S)

•Count of tablets of betahistine taken

•Record of new medication taken, for any cause, from visit 1

•Registration of days of temporary disability during the previous year caused by vertigo or any other cause, in employed participants

•Record of medical consultations due to dizziness.

### Statistical analysis

Data will be analyzed in accordance with the CONSORT guide for cluster randomized trials, and all analyses will be performed on an intent-to-treat basis [28].

Firstly, the intervention group and control group will be analyzed for baseline comparability according to the baseline variables. Descriptive statistics of all studied variables will be presented in contingency tables. Pearson’s Chi-square test or Fisher’s exact test will be applied to assess categorical variables. Student’s *t-*test or ANOVA will be used if the variables follow a normal distribution and Mann–Whitney *U*-test if they do not (objectives 1 and 2).

A multilevel logistic regression analysis will be performed to evaluate the association between the dependent variable (cured/not cured) and the independent variable (group assigned), adjusting potential confounders.

A multivariate linear regression model will be conducted to evaluate change in the Likert scale and quality of life inventory (DHI-S), (objectives 5 and 6); and a Poisson or Binomial negative distribution (for of outcome variables in case of over-dispersion) will be used to compare number of vertigo episodes, number of tablets consumed, days of temporary disability and days with symptoms (objectives 3, 7 and 8).

In order to evaluate D-H test’s intra- and inter-observer variations, corresponding Kappa ponderate indexes (Kp) and intra-class correlation coefficients (ICC) will be calculated. Intra-observer variations will be determined through comparison of the D-H test’s results, performed twice on each patient, after a five-minute interval, for a total number of ten patients per observer, in any visit. Inter-observer variations will be determined through comparison of the D-H test’s results on each patient, conducted by pairs of observers, after a five-minute interval, for a total number of ten patients per pair of observers, in any visit. The estimated number of observers will be 10 to 12. Homogeneity and concordance in the Epley’s maneuver performance will be evaluated by an expert external auditor, who will assess between two and three video-registered maneuvers carried out by each observer.

We will apply the Cox regression model to explore the effect of the intervention (Epley’s maneuver) on the survival (that is interval between two vertigo episodes) (objective 4).

Statistical analysis will be carried out by statisticians from the Institut Universitari d’Investigació en Atenció Primària Jordi Gol (IDIAP Jordi Gol), who will have an advisory role. Data will be introduced into a SPSS version 18.0 system database (SPSS Inc, Chicago, IL, USA). Descriptive analysis, and statistical hypothesis testing, will also be conducted with the SPSS version 18.0 system, with blinding. The level of statistical significance will be set at 0.05 and all tests will be two-tailed. All known potential confounding factors will be measured at the beginning of the study and comparison between the control and the intervention groups will be carried out adjusted for these known confounders.

### Ethical aspects

The protocol has been reviewed and approved by the CEIC (Clinical Research Ethics Committee) of IDIAP Jordi Gol, with the number P12/69. Obtaining a signed informed consent from the participants will be a mandatory requirement before study initiation. Study information will be provided verbally and in writing to all participants. Participants in the study will have the opportunity to resolve any doubts about study details. The written consent states that the study follows the law contained in the Helsinki Declaration and in Title I, Article 12 of the Royal Spanish Decree 561/1993 from 16 April 1993.

Data confidentiality: participants will be informed that data will be treated with absolute confidentiality according to the organic law that regulates the confidentiality of computerized data (Organic law 5/1992), and that data will be used exclusively for the objectives of the study.

## Discussion

Our study aims to demonstrate the effectiveness of repositioning maneuvers in the treatment of posterior canal BPPV, performed by trained GPs in the primary care setting. We found that only one study had been conducted in primary care, and it proved that this treatment was effective in the first week, regarding negativization of D-H test, but not in terms of subjective improvement of patients [18]. This study only evaluated patients from baseline to week 1. In our study we will reassess patients one week, one month and one year after the first visit.

### Study limitations

Frentzel glasses (a diagnostic tool to evaluate nystagmus) were not used in the study in order to achieve a more realistic approach in the diagnosis of BPPV in primary care. GPs’ lack of experience in ENT skills may reduce reliability of the maneuvers but it resembles usual practice in primary care settings. Therefore, study patients who only experience vertigo and do not present nystagmus during the D-H test will not be excluded from inclusion (as performed in other studies) [29]. As we mentioned on the D-H test results section, we will consider both vertigo with nystagmus and vertigo without nystagmus as positive D-H results; the reason being that GPs’ lack of experience in ENT skills, and not using the Frentzel glasses may diminish the D-H test sensitivity by missing less clear cases of nystagmus. This factor, however, will be accounted for by evaluating these patients separately.

Betahistine administration during the course of treatment can speed up the recovery of these patients [25,26] and may also be useful to evaluate the results of the study. To avoid bias in our trial, all patients from both groups (intervention and control), will be prescribed betahistine 8 mg on a *pro re nata* (PRN) basis, up to three times a day until improvement of symptoms. Each participant will be given a notebook in order to record the number of tablets taken between visits. From an ethical point of view, we believe that leaving patients untreated, apart from the sham maneuver is an arguable point.

The incidence of BPPV may hinder securing an adequate sample size. However, internal data from computerized clinical records indicates that this disorder is much more frequent than previously reported in the literature. Moreover, BPPV prevalence increases with age, reaching 10% in individuals over 80 years old [4].

As this trial evaluates a therapeutic maneuver, the blinding achieved in this study may not be comparable to that of a double-blind pharmacological trial. On the other hand, GPs may present lower skills than experienced ENT specialists, in assessing nystagmus, performing the D-H test and the Epley’s repositioning maneuver. Even though some authors support that GPs are qualified to perform these skills [29], this factor will be controlled measuring the inter-observer and intra-observer variability for each maneuver and evaluation, and including ‘observer’ as a potential confounder in a multilevel logistic regression.

A reduced ability in the performance of Epley’s maneuver would decrease its effectiveness in our patients, and may counter to the hypothesis of this trial. Despite all GPs participating in the trial having been trained in performing the maneuvers, inclusion of false positive cases may not be ruled out, with a subsequent decline in the observed effect.

As in any follow-up study, loss of participants over time may occur. In order to diminish loss to follow-up, we will introduce telephone reminders before patient appointments and will establish contact with their GPs, to help recruitment if the latter fails.

### Strengths of the study

This project aims to improve quality of life in patients with BPPV by implementing a safe, simple and effective technique which may avoid unnecessary laboratory tests, extensive additional testing, referral to specialists and longer temporary disability.

Due to the lack of use of the D-H test, and Epley’s maneuver by GPs, most of these patients are not correctly diagnosed or treated, [20-22], and consequently suffer from a longer duration of their symptoms and disability. Moreover, they are frequently treated with drugs, often for long periods of time, with subsequent unnecessary prolonged side effects and expense.

Positive results in our study would highlight the significance of these techniques in primary care and may encourage GPs to implement them in their usual practice. Moreover, they would enable the development of new guidelines and models for the interoperability between primary care and ENT specialists.

## Trial status

The status of the trial at the time of manuscript submission is recruiting patients.

## Abbreviations

BOE: Boletín oficial del Estado; BPPV: benign paroxysmal positional vertigo; CAP: Centro de Atención Primaria; CEIC: Comité Ético de Investigación Clínica; CI: confidence interval; CONSORT: Consolidated Standards of Reporting Trials; CRF: case report form; CRP: Canalith Repositioning Procedure; DAP: Dirección de Atención Primaria; DH: Dix-Hallpike; DHI: Dizziness Handicap Inventory; DHI-S: Dizziness Handicap Inventory-Short form; EAP: Equipo de Atención Primaria; ENTs: otolaryngologists; GPs: general practitioners; ICC: intra-class correlation coefficients; ICS: Institut Català de la Salut; IDIAP Jordi Gol: Institut Universitari d’Investigació en Atenció Primària Jordi Gol; ITT: intent-to-treat; Kp: Kappa ponderate indexes; OR: odds ratio; ORL: Otorrinolaringología; PRN: ‘*pro re nata*’ (Latin: as needed); REAP: Red Española de Atención Primaria; SAP: Servicio de Atención Primaria; UGEAP: Unidad de Gestión de Atención Primaria.

## Competing interests

The study authors declare that they have no competing interests.

## Authors’ contributions

JLBM: conception, design and drafting, data collection, manuscript writing and final approval of the manuscript. RCM: conception, design and drafting, data collection, manuscript writing and final approval of the manuscript. IVB: data collection, manuscript writing, analysis and interpretation of data, critical revision and final approval of manuscript. YRM: data collection and analysis, critical revision and final approval of manuscript. OLAA: data collection and analysis, critical revision and final approval of manuscript. AV: data collection and analysis, critical revision, translation of the manuscript into English and its final approval. OBA: data collection and analysis, critical revision and final approval of manuscript. JAO: conception, design and drafting, analysis and interpretation of data, manuscript writing and final approval of manuscript. ACG: data collection and analysis, critical revision and final approval of manuscript. CJBO: data collection and analysis, critical revision and final approval of manuscript. PCSP: data collection and analysis, critical revision and final approval of manuscript. XMC: data collection and analysis, critical revision and final approval of manuscript. ERP: data collection and analysis, critical revision and final approval of manuscript. CRR: data collection and analysis, critical revision and final approval of manuscript. NMF: data collection and analysis, critical revision and final approval of manuscript. AMHS: interpretation of data, critical revision, in charge of overseeing the training of all researchers of the Dix-Hallpike and final approval of manuscript. CMC: analysis and interpretation of data, critical revision and final approval of manuscript. RAL: analysis and interpretation of data, critical revision and final approval of manuscript. All authors read and approved the final manuscript.

## Authors’ information

JLBM, RCM, IVB, YRN, OLAA, OBA, XMC, ERP, CRR, NMF, CMC, RAL, AV, ACG, CJBO, PCSP are GPs; and AMHS is an ENT specialist.

## Supplementary Material

Additional file 1: Video 1 Dix-Hallpike maneuver. Click here for file

Additional file 2: Video 2 Epley’s maneuver. Click here for file
